# Docosahexaenoic and eicosapentaenoic acids increase prion formation in neuronal cells

**DOI:** 10.1186/1741-7007-6-39

**Published:** 2008-09-12

**Authors:** Clive Bate, Mourad Tayebi, Luisa Diomede, Mario Salmona, Alun Williams

**Affiliations:** 1 Department of Pathology and Infectious Diseases, Royal Veterinary College, Hawkshead Lane, North Mymms, Herts, UK. AL9 7TA; 2 Department of Molecular Biochemistry and Pharmacology, Istituto di Ricerche Farmacologiche 'Mario Negri', Via La Masa, 20156, Milan, Italy

## Abstract

**Background:**

The transmissible spongiform encephalopathies, otherwise known as prion diseases, occur following the conversion of the cellular prion protein (PrP^C^) to an alternatively folded, disease-associated isoform (PrP^Sc^). Recent studies suggest that this conversion occurs via a cholesterol-sensitive process, as cholesterol synthesis inhibitors reduced the formation of PrP^Sc ^and delayed the clinical phase of scrapie infection. Since polyunsaturated fatty acids also reduced cellular cholesterol levels we tested their effects on PrP^Sc ^formation in three prion-infected neuronal cell lines (ScGT1, ScN2a and SMB cells).

**Results:**

We report that treatment with docosahexaenoic acid (DHA), eicosapentaenoic acid (EPA) or the cholesterol synthesis inhibitor simvastatin reduced the amounts of free cholesterol in membrane extracts from prion-infected neuronal cells. Simvastatin reduced cholesterol production while DHA and EPA promoted the conversion of free cholesterol to cholesterol esters. Crucially, while simvastatin reduced PrP^Sc ^formation, both DHA and EPA significantly increased the amounts of PrP^Sc ^in these cells. Unlike simvastatin, the effects of DHA and EPA on PrP^Sc ^content were not reversed by stimulation of cholesterol synthesis with mevalonate. Treatment of ScGT1 cells with DHA and EPA also increased activation of cytoplasmic phospholipase A_2 _and prostaglandin E_2 _production. Finally, treatment of neuronal cells with DHA and EPA increased the amounts of PrP^C ^expressed at the cell surface and significantly increased the half-life of biotinylated PrP^C^.

**Conclusion:**

We report that although treatment with DHA or EPA significantly reduced the free cholesterol content of prion-infected cells they significantly increased PrP^Sc ^formation in three neuronal cell lines. DHA or EPA treatment of infected cells increased activation of phospholipase A_2_, a key enzyme in PrP^Sc ^formation, and altered the trafficking of PrP^C^. PrP^C ^expression at the cell surface, a putative site for the PrP^Sc ^formation, was significantly increased, and the rate at which PrP^C ^was degraded was reduced. Cholesterol depletion is seen as a potential therapeutic strategy for prion diseases. However, these results indicate that a greater understanding of the precise relationship between membrane cholesterol distribution, PrP^C ^trafficking, cell activation and PrP^Sc ^formation is required before cholesterol manipulation can be considered as a prion therapeutic.

## Background

Transmissible spongiform encephalopathies (TSEs), also known as prion diseases, include Creutzfeldt-Jakob disease and kuru in humans, scrapie in sheep and goats, and bovine spongiform encephalopathy in cattle. The central event in these diseases is thought to be the conversion of a host-encoded cellular prion protein (PrP^C^) into an abnormally folded disease-associated isoform, designated PrP^Sc ^[1]. Aggregates of PrP^Sc ^accumulate around neurons in affected brain areas [2], a process which is thought to lead to neuronal dysfunction and the clinical symptoms of infection. PrP^Sc ^constitutes the major and perhaps the only component of the infectious particle [3].

The process of prion replication has been studied extensively in prion-infected neuronal cell lines. Treatment with some cholesterol synthesis inhibitors reduced the production of PrP^Sc ^in scrapie-infected neuronal cells [4-6]. The anti-prion effect of such drugs is attributed to cholesterol depletion affecting the formation of specialised membrane micro-domains called lipid rafts [7]. These lipid rafts are highly enriched in cholesterol, sphingolipids and gangliosides, and contain specific proteins [8]. The presence of a glycosylphosphatidylinositol (GPI) anchor that mediates the attachment of proteins including PrP^C ^and PrP^Sc ^to membranes, targets these proteins to lipid rafts [9].

Since cholesterol levels are a factor determining PrP^Sc ^formation [4-6], the effects of compounds reported to affect cellular cholesterol levels were examined. Polyunsaturated fatty acids (PUFA) are fatty acids which contain two or more double bonds within their hydrocarbon chain. They are taken as dietary supplements by large numbers of people for their perceived health benefits against a variety of diseases including coronary heart disease, hypertension, diabetes mellitus and rheumatoid arthritis [10]. The common PUFA include docosahexaenoic acid (DHA), eicosapentaenoic acid (EPA), arachidonic acid (AA), linoleic acid (LA) and linolenic acid (LNA) and in most cells PUFA are rapidly incorporated into phospholipids [11]. The incorporation of PUFA into phospholipids alters the composition and physical properties of cell membranes [12]. Since dietary PUFA reduce cellular cholesterol levels [13] the effects of PUFA on the composition of neuronal cell membranes and on the production of PrP^Sc ^were examined. We report that treatment with DHA or EPA significantly reduced the amounts of free cholesterol in ScGT1, ScN2a and SMB cells. However, in contrast to the effects of cholesterol synthesis inhibitors, treatment with DHA or EPA actually increased PrP^Sc ^formation. DHA or EPA treatment also increased the activity of phospholipase A_2 _(PLA_2_), an enzyme reported to affect PrP^Sc ^formation [14]. They also increased the amount of PrP^C^, that is essential for the development of prion diseases [15,16], at the cell surface, a putative site for PrP^Sc ^formation. In conclusion our data indicate that cholesterol depletion *per se *does not reduce PrP^Sc ^formation.

## Results

### DHA and EPA increase PrP^Sc ^in prion-infected neuronal cells

**Figure 1:**
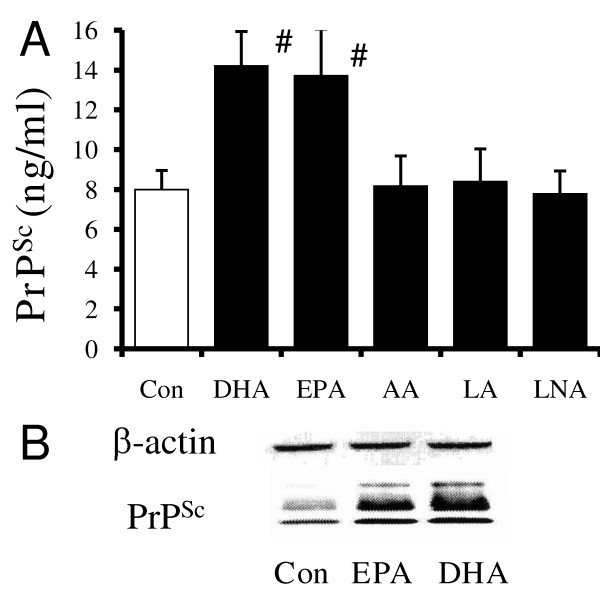
**Treatment with DHA or EPA increases the PrP^Sc ^content of ScGT1 cells**. (A) The amounts of PrP^Sc ^in ScGT1 cells treated for 7 days with 1 μM PUFA as shown (■) or with control medium (□). Values shown are the mean average amounts of PrP^Sc ^(ng/ml) ± SD, *n *= 15. # = PrP^Sc ^content significantly higher than that of control cells. (B) Immunoblots showing the amounts of β-actin and PrP^Sc ^in extracts from ScGT1 cells treated with control medium or 1 μM DHA or EPA as shown.

**Figure 2:**
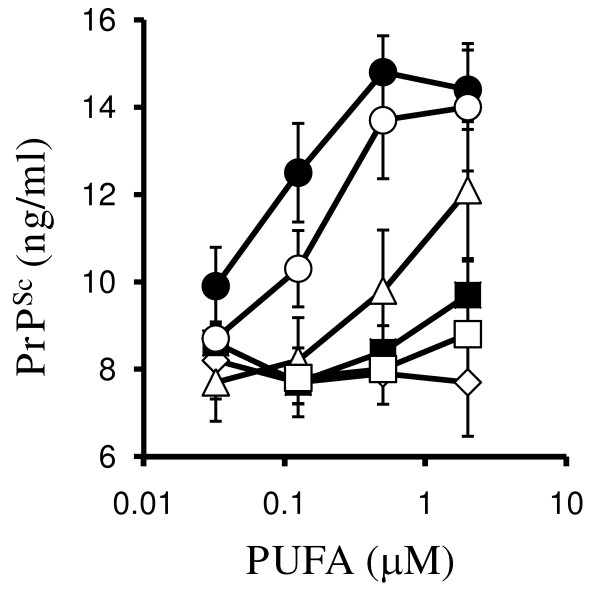
**Dose-dependent effects of PUFA on PrP^Sc ^formation**. The amounts of PrP^Sc ^in ScGT1 cells treated for 7 days with different concentrations of DHA (●), EPA (○), DPA (△), LA (■), DTA (□) or ETA (◇). Values shown are the mean average amounts of PrP^Sc ^(ng/ml) ± SD, *n *= 9.

The effect of PUFA on the formation of PrP^Sc ^in ScGT1 cells was determined by daily treatment with 1 μM PUFA for 7 days. Significantly higher amounts of PrP^Sc ^were found in cells treated with DHA (14.2 ng/ml ± 1.7 compared with 8 ± 1, *n *= 15, *P *= 0.00000000001) or EPA (13.8 ng/ml ± 2.4 compared with 8 ± 1, *n *= 15, *P *= 0.00000002) than in untreated cells, or cells treated with AA, LA or LNA (Figure 1A). Treatment with DHA or EPA did not affect the survival of ScGT1 cells (data not shown). Immunoblots were used to verify the ELISA data; these showed that there were observable differences in the amounts of PrP^Sc ^between untreated cells and cells treated with 1 μM DHA or 1 μM EPA (Figure 1B). In contrast, the amounts of β-actin in untreated cells and cells treated with DHA or EPA were not significantly different. Determination of the effects of different PUFA on PrP^Sc ^formation in ScGT1 cells revealed that whereas treatment with either DHA or docosapentaenoic (DPA) significantly increased amounts of PrP^Sc ^present, treatment with docosatetraenoic acid (DTA) or LA did not. Similarly, while treatment with EPA increased the amounts of PrP^Sc ^in ScGT1 cells, treatment with eicosatetraenoic acid (ETA) did not. The effects of DHA, DPA and EPA on the PrP^Sc ^content of ScGT1 cells were dose-dependent (Figure 2).

To confirm that the effects of DHA or EPA were not specific for ScGT1 cells the amounts of PrP^Sc ^in ScN2a and SMB cells were also examined. The amounts of PrP^Sc ^in ScN2a cells treated for 7 days with 1 μM DHA or 1 μM EPA, but not in cells treated with LNA, LA or AA, were significantly higher than in untreated cells (Table 1). Similar results were obtained with SMB cells; treatment with DHA or EPA increased the amounts of cell-associated PrP^Sc^. Since PrP^Sc ^can be released from cells as exosomes or as result of cell damage [17], the amounts of PrP^Sc ^in the supernatants of treated cells were also measured. The amounts of PrP^Sc ^in the supernatants of ScGT1, ScN2a or SMB cells treated with 1 μM DHA or 1 μM EPA were significantly higher than in supernatants from untreated cells (Table 1).

**Table 1:** Effects of PUFA on cell-associated and secreted PrP^Sc^

	**PrP^Sc ^(ng/ml)**					
**Treatment**	**ScGT1 cells**		**ScN2A cells**		**SMB cells**	

	**Cell extracts**	**Supernatant**	**Cell extracts**	**Supernatant**	**Cell extracts**	**Supernatant**

**None**	8.0 ± 1.0	1.42 ± 0.12	1.38 ± 0.14	0.45 ± 0.09	6.2 ± 1.1	3.1 ± 0.8
**1 μM DHA**	14.2 ± 1.7 *	4.64 ± 0.73 *	2.91 ± 0.71 *	1.12 ± 0.12 *	16.4 ± 3.7 *	5.7 ± 3.1 *
**1 μM EPA**	13.8 ± 2.4 *	3.42 ± 0.6 *	3.58 ± 0.52 *	0.96 ± 0.25 *	14.1 ± 2.2 *	4.8 ± 1.2 *
**1 μM LA**	8.4 ± 1.6	1.38 ± 0.21	1.43 ± 0.2	0.68 ± 0.4	7.7 ± 2.5	2.6 ± 0.75
**1 μM LNA**	7.8 ± 1.1	1.54 ± 0.46	1.63 ± 0.31	0.51 ± 0.22	7.1 ± 1.8	2.1 ± 1.15
**1 μM AA**	8.1 ± 1.5	1.56 ± 0.3	1.44 ± 0.3	0.59 ± 0.11	6.4 ± 1.5	2.5 ± 1.26

The amounts of PrP^Sc ^in cell extracts (10^6 ^cells/ml) and in culture supernatants (equivalent to 10^6 ^cells/ml) from prion-infected neuronal cells treated for 7 days with 1 μM PUFA as shown. Amounts of PrP^Sc ^were measured by PrP-specific ELISA and are expressed as the mean average PrP^Sc ^(ng/ml) ± SD of ScGT1 (*n *= 15), ScN2a (*n *= 10) and SMB cells (*n *= 9). * = amounts of PrP^C ^significantly different from that of untreated cells (*P *< 0.01).

### DHA and EPA reduced free cholesterol in ScGT1 cells

The effect of 24-hour exposure to PUFA on the cholesterol content of ScGT1 cell extracts was examined. Exposure to 1 μM DHA or EPA, but not to LNA or LA, significantly reduced their free cholesterol content (Table 2). None of the PUFA tested had a significant effect on the amounts of protein in these extracts (data not shown). The amounts of free cholesterol in cells treated with DHA or EPA were similar to those in cells treated with 5 μM simvastatin, which inhibits 3-hydroxy-3-methylglutaryl-CoA (HMG-CoA) reductase, the rate-limiting step in cholesterol synthesis [18]. However, total cholesterol is a combination of free cholesterol found in cell membranes and cholesterol esters. Simvastatin reduced the amounts of free cholesterol in cell extracts without affecting the amounts of cholesterol esters, resulting in a reduction in total cellular cholesterol. In contrast, the total cholesterol content of ScGT1 cells treated with DHA or EPA was not significantly altered, as the reduction in free cholesterol was accompanied by significant increases in the amounts of cholesterol esters. The addition of DHA or EPA to ScN2a or SMB cells had similar effects; they significantly reduced free cholesterol levels and increased the cholesterol ester content of these cells (data not shown).

**Table 2:** Effects of PUFA on the distribution of cholesterol in ScGT1 cells

**Treatment**	**Cholesterol ng/ml**		
	**Total**	**Free**	**Esterified**

**None**	590 ± 54	515 ± 29	75 ± 26
**DHA (1 μM)**	544 ± 39	411 ± 26 #	133 ± 31 @
**EPA (1 μM)**	553 ± 28	438 ± 25 #	115 ± 20 @
**LNA (1 μM)**	607 ± 26	548 ± 43	59 ± 18
**LA (1 μM)**	572 ± 74	495 ± 52	77 ± 28
**Simvastatin (5 μM)**	438 ± 46 *	354 ± 41 #	84 ± 32
**Cholesterol myristate (10 μM)**	762 ± 55 *	538 ± 51	224 ± 41 @
**Cholesterol arachidonate (10 μM)**	734 ± 47 *	545 ± 38	189 ± 32 @

The amounts of total, free and esterified cholesterol in extracts from ScGT1 cells treated for 24 hours with PUFA, simvastatin, or with cholesterol esters as shown. Amounts of total, free and esterified cholesterol were expressed as the mean average (ng/ml) ± SD, *n *= 9. Amounts of total cholesterol (*), free cholesterol (#) or esterified cholesterol (@) significantly different from that of untreated cells (*P *< 0.01).

Such observations raised the possibility that the increased amounts of cholesterol esters in prion-infected cells facilitate PrP^Sc ^formation. This possibility was examined by treating ScGT1 cells with 10 μM cholesterol esters (cholesterol myristate or cholesterol arachidonate). We report that although treatment increased the amounts of cholesterol esters in ScGT1 cells, there were no significant differences in the amounts of free cholesterol in these cells (Table 2), indicating that the cholesterol esters were not hydrolysed. The addition of cholesterol esters did not affect PrP^Sc ^formation; there were no significant differences between the PrP^Sc ^content of vehicle-treated ScGT1 cells and ScGT1 cells treated for 7 days with 10 μM cholesterol myristate (8.1 ng/ml ± 1.2 compared with 7.9 ± 1.0, *n *= 8, *P *= 0.8) or cholesterol arachidonate (8.1 ng/ml ± 1.2 compared with 8.5 ± 1.7, *n *= 8, *P *= 0.6). Similarly, the addition of cholesterol myristate or cholesterol arachidonate did not significantly affect the amounts of PrP^Sc ^in ScN2a cells or in SMB cells (data not shown).

### Effects of DHA and EPA are not reversed by mevalonate

**Figure 3:**
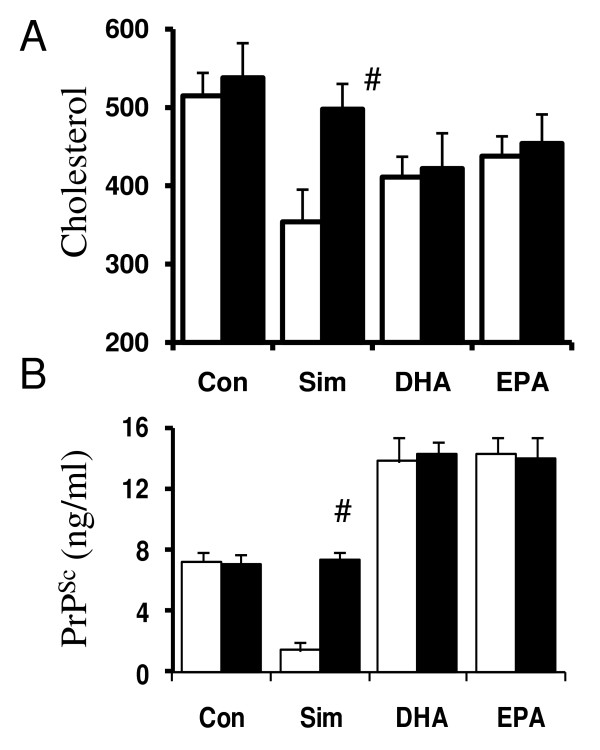
**The effects of DHA or EPA are not reversed by mevalonate**. (A) The amounts of free cholesterol in extracts from ScGT1 cells treated for 7 days with control medium, 5 μM simvastatin (Sim), 1 μM DHA or 1 μM EPA as shown (□), and with the addition of 100 μM mevalonate (■). Values shown are the mean average amounts of free cholesterol (ng/ml) ± SD, *n *= 9. # = amounts of free cholesterol significantly higher following the addition of mevalonate. (B) The amounts of PrP^Sc ^in ScGT1 cells treated for 7 days with control medium, 5 μM simvastatin (Sim), 1 μM DHA, 1 μM EPA as shown (□) and with the addition of 100 μM mevalonate (■). Values shown are the mean average amounts of PrP^Sc ^(ng/ml) ± SD, *n *= 9. # = amounts of PrP^Sc ^significantly higher following the addition of mevalonate.

The effects of DHA and EPA on cellular cholesterol levels and PrP^Sc ^formation were compared with those of simvastatin. As shown in Figure 3A, the exposure of ScGT1 cells to 1 μM DHA or EPA for 7 days significantly decreased the free cholesterol content of cell extracts, as did 5 μM simvastatin. However, while the addition of 100 μM mevalonate significantly increased the amounts of free cholesterol in simvastatin-treated cells (521 free cholesterol (ng/ml) ± 45 compared with 356 ng/ml ± 49, *n *= 9, *P *= 0.0002), it did not affect the amounts of free cholesterol in cells treated with DHA (423 ng/ml ± 40 compared with 415 ± 43, *n *= 9, *P *= 0.4) or EPA (451 ng/ml ± 37 compared with 428 ± 38, *n *= 9, *P *= 0.17). Rather, the addition of mevalonate to DHA-treated cells significantly increased the amounts of cholesterol esters (225 ng/ml ± 28 compared with 133 ± 31, *n *= 9, *P *< 0.01). Next we examined the effects of mevalonate on PrP^Sc ^formation in ScGT1 cells treated with simvastatin, DHA or EPA. The addition of 100 μM mevalonate significantly increased the amounts of PrP^Sc ^in simvastatin-treated cells (7.4 ng/ml ± 0.5 compared with 1.4 ± 0.9, *n *= 9, *P *= 0.00002). In contrast, it did not affect the amounts of PrP^Sc ^in ScGT1 cells treated with DHA (13.8 ng/mg ± 1.5 compared with 14.3 ± 0.9, *n *= 9, *P *= 0.4) or EPA (14.3 ng/mg ± 1.1 compared with 13.9 ± 1.5, *n *= 9, *P *= 0.8), indicating that the effect of DHA and EPA on PrP^Sc ^formation was not due to HMG-CoA reductase inhibition (Figure 3B).

### DHA increases PLA_2 _activity of prion-infected neuronal cells

**Figure 4:**
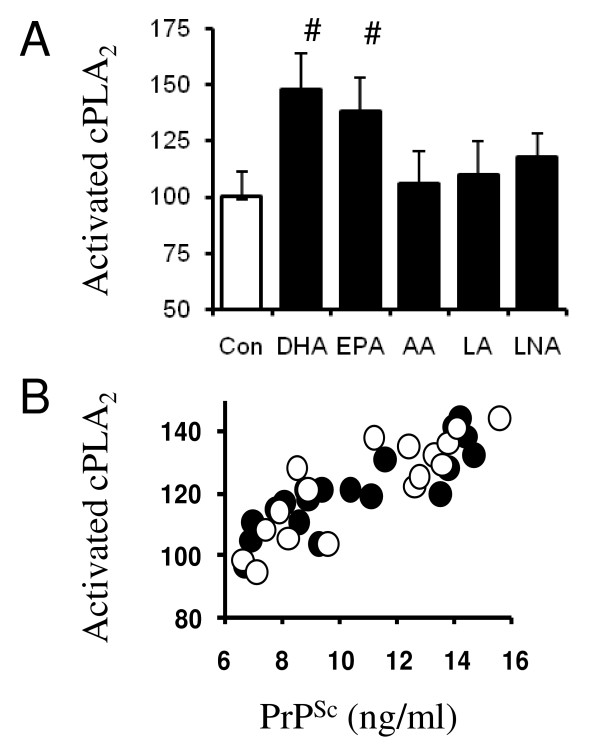
**DHA and EPA increases the amounts of activated cPLA_2 _in ScGT1 cells**. (A) The amounts of activated (phosphorylated) cPLA_2 _in untreated ScGT1 cells (□) or ScGT1 cells treated with 1 μM PUFA as shown (■). Values shown are the mean average amounts of activated cPLA_2 _(units) ± SD, *n *= 12. # = amounts of activated cPLA_2 _significantly higher than that of untreated cells. (B) The amounts of activated cPLA_2 _(units) in ScGT1 cells treated for 24 hours with different concentrations of DHA or EPA were plotted against the amounts of PrP^Sc ^(ng/ml) in ScGT1 cells treated for 7 days with the same concentrations of DHA (●) or EPA (○).

Since the amounts of activated cytoplasmic PLA_2 _(cPLA_2_) in ScGT1 cells correlates with the amounts of PrP^Sc ^[19] and PLA_2 _inhibitors reduce PrP^Sc ^formation [14], the effects of PUFA on the activity of PLA_2 _in ScGT1 cells was determined. We report that treatment with 1 μM DHA for 24 hours significantly increased the amounts of phosphorylated (activated) cPLA_2 _in ScGT1 cells (152 units activated cPLA_2 _± 15 compared with 100 ± 9, *n *= 9, *P *= 0.00001). Treatment with 1 μM EPA for 24 hours also significantly increased amounts of activated cPLA_2 _(142 units ± 13 compared with 100 ± 9, *n *= 9, *P *= 0.0001), but treatment with 1 μM AA, LA or LNA had no significant affect (Figure 4A). The addition of 10 μM cholesterol myristate did not affect activation of cPLA_2 _in ScGT1 cells (100 units ± 11 compared with 98 ± 8, *n *= 9, *P *= 0.93). This effect of treatment with 1 μM DHA or EPA was specific for prion-infected cells and did not affect the amounts of activated cPLA_2 _in GT1 cells or primary cortical neurons. To examine this relationship further we compared the amounts of PrP^Sc ^and activated cPLA_2 _in ScGT1 cells treated with varying concentrations of DHA or EPA. There was a positive correlation between the amounts of activated cPLA_2 _at 24 hours and the PrP^Sc ^content of cells after 7 days (Pearson coefficient = 0.847, *P *< 0.001) (Figure 4B).

**Figure 5:**
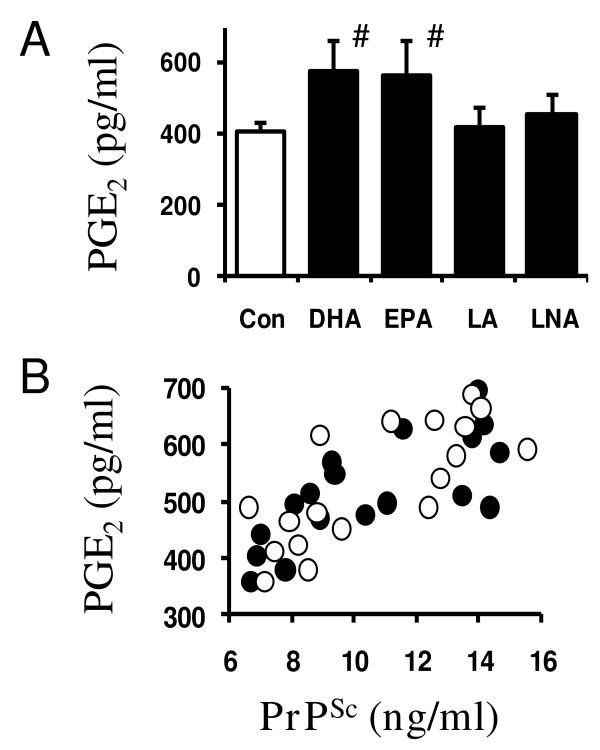
**DHA and EPA increase the amounts of PGE_2 _in ScGT1 cells**. (A) The amounts of PGE_2 _produced by untreated ScGT1 cells (□) or by ScGT1 cells treated for 24 hours with 1 μM PUFA as shown (■). Values shown are the mean average amounts of PGE_2 _(pg/ml) ± SD, *n *= 9. # = PGE_2 _content significantly higher than that of control cells. (B) The amounts PGE_2 _(pg/ml) in ScGT1 cells treated for 24 hours with different concentrations of DHA or EPA were plotted against the amounts of PrP^Sc ^(ng/ml) in ScGT1 cells treated for 7 days with the same concentrations of DHA (●) or EPA (○).

The cPLA_2 _isoform is largely responsible for the release of AA and increased production of prostaglandins. Here we show that the amounts of prostaglandin E_2 _(PGE_2_) produced by ScGT1 cells was significantly increased by 24-hour treatment with 1 μM DHA (577 ± 89 pg/ml compared with 407 ± 51, *n *= 9, *P *= 0.0002) or EPA (568 ± 98 pg/ml compared with 407 ± 51, *n *= 9, *P *= 0.001), but not by LA or LNA (Figure 5A). The addition of 1 μM DHA or EPA did not affect PGE_2 _production in GT1 cells. Furthermore, the increased amounts of PrP^Sc ^in DHA or EPA-treated ScGT1 cells showed a positive correlation with the amounts of PGE_2 _produced (Pearson's coefficient = 0.749, *P *< 0.001; Figure 5B). Treatment with 1 μM DHA also increased the production of PGE_2 _in ScN2a cells (319 pg/ml ± 29 compared with 220 pg/ml ± 24, *n *= 6, *P *< 0.01) and in SMB cells (487 pg/ml ± 89 compared with 355 pg/ml ± 44, *n *= 6, *P *< 0.01).

### DHA increases the surface expression of PrP^C^

Since PrP^C ^is necessary for PrP^Sc ^formation [15,16]. the effects of PUFA treatment on the expression of PrP^C ^were examined. These studies were initially performed on primary cortical neurons that do not contain the infectious PrP^Sc ^to avoid any possible confusion between PrP^C ^and PrP^Sc^. Treatment with 1 μM PUFA for 24 hours did not affect the total amounts of PrP^C ^in whole cell membrane extracts. Although PrP^C ^is normally found within lipid raft micro-domains [9], treatment with 2 μM simvastatin caused the redistribution of PrP^C ^into the normal bulk membrane. In contrast, treatment with PUFA did not alter the distribution of PrP^C ^between lipid rafts and the bulk membrane (Table 3).

**Table 3:** Treatment with PUFA does not affect the amounts of PrP^C ^in neuronal membranes

**Treatment**	**PrP^C ^(ng/ml)**		
	**Whole cell extract**	**Lipid raft fraction**	**Bulk fraction**

**None**	31.6 ± 4.8	25.5 ± 2	2.9 ± 0.9
**1 μM AA**	30.2 ± 3.1	26.8 ± 2.9	2.8 ± 0.5
**1 μM DHA**	33.2 ± 4.4	26.6 ± 3.4	3.8 ± 0.8
**1 μM EPA**	34.5 ± 2.9	27.8 ± 3.1	3.1 ± 0.3
**1 μM LA**	29.9 ± 4.1	25.9 ± 2.8	3.4 ± 0.8
**1 μM LNA**	28.8 ± 4.7	24.5 ± 2	3.5 ± 1.2
**2 μM Simvastatin**	35.6 ± 4.2	7.2 ± 2.2 *	25.9 ± 3.8 *

The amounts of PrP^C ^in whole cell extracts, detergent insoluble membranes (lipid raft fraction) and the detergent soluble membrane (bulk fraction) from primary cortical neurons treated with 1 μM PUFA or 2 μM simvastatin for 24 hours. Amounts of PrP^C ^were measured by ELISA and are expressed as the mean average PrP^C ^(ng/ml) ± SD, *n *= 12. * = amounts of PrP^C ^significantly different from that of untreated cells (*P *< 0.01).

**Figure 6:**
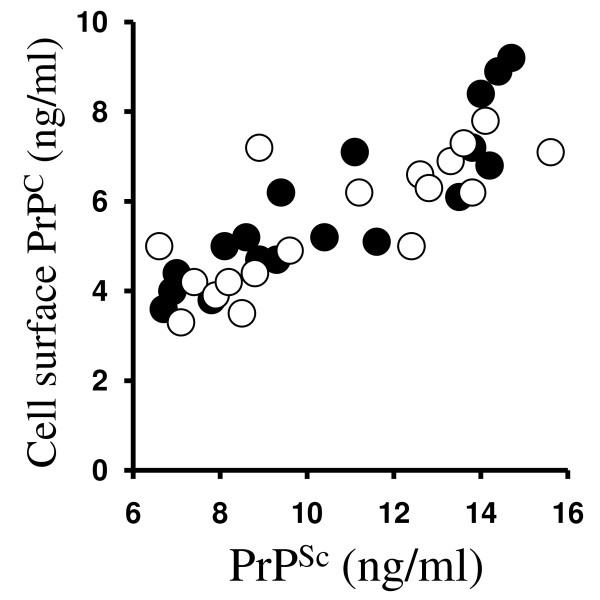
**DHA and EPA increase the amounts of PrP^C ^at the surface of ScGT1 cells**. The amounts of PrP^C ^(ng/ml) released from the surface of ScGT1 cells treated for 24 hours with different concentrations of DHA or EPA were plotted against the amounts of PrP^Sc ^(ng/ml) in ScGT1 cells treated for 7 days with the same concentrations of DHA (●) or EPA (○).

The amounts of PrP^C ^at the cell surface were measured by digesting neurons with phosphatidylinositol-specific phospholipase C (PI-PLC). PI-PLC digestion releases GPI-anchored molecules including PrP^C ^from the cell surface [20]. The amounts of PrP^C ^released by PI-PLC from 10^6 ^cortical neurons treated with 1 μM DHA or 1 μM EPA for 24 hours were significantly higher than the amounts released from untreated neurons or neurons treated with 10 μM cholesterol esters (Table 4). Treatment with DHA or EPA for 24 hours also significantly increased the amounts of surface PrP^C ^in GT1 and ScGT1 cells. It is noteworthy that the amounts of PrP^C ^released by PI-PLC from untreated ScGT1 cells were significantly higher than from untreated GT1 cells (4.1 ng/ml ± 0.8 compared with 1.8 ± 0.6, *n *= 12, *P *< 0.01). The effect of DHA or EPA on cell surface PrP^C ^expression contrasted to that of simvastatin, which reduced surface PrP^C ^expression [21]. When the amounts of PrP^C ^at the surface of ScGT1 cells treated with different concentrations of DHA or EPA were plotted against the amounts of PrP^Sc ^in ScGT1 treated for 7 days with the same concentrations of DHA or EPA, a significant correlation was observed (Pearson coefficient = 0.832, *P *< 0.001) (Figure 6).

**Table 4:** Treatment with DHA or EPA increases the amounts of PrP^C ^at the cell surface

**Treatment**	**Cell surface PrP^C ^(ng/10^6 ^cells)**		
	**Cortical Neurons**	**GT1 cells**	**ScGT1 cells**

**None**	2.9 ± 0.4	1.8 ± 0.6	4.1 ± 0.8
**1 μM AA**	3.1 ± 0.4	2.1 ± 0.4	3.7 ± 1.1
**1 μM DHA**	4.8 ± 0.4 ^#^	3.1 ± 0.6 ^#^	8.6 ± 1.6 ^#^
**1 μM EPA**	4.5 ± 0.5 ^#^	2.8 ± 0.5 ^#^	6.7 ± 1.2 ^#^
**1 μM LA**	3.4 ± 0.6	2.5 ± 0.6	5 ± 0.7
**1 μM LNA**	3.2 ± 0.6	2.3 ± 0.5	5.8 ± 0.8 ^#^
**2 μM Simvastatin**	0.6 ± 0.3 *	0.5 ± 0.2 *	0.7 ± 0.4 *

The amounts of PrP^C ^at the surface of 10^6 ^cells treated with 1 μM PUFA or 2 μM simvastatin for 24 hours were measured following digestion with PI-PLC (0.2 units/ml). Amounts of PrP^C ^were measured by ELISA and are expressed as the mean average PrP^C ^(ng/10^6 ^cells) ± SD, *n *= 12. ^# ^= amounts of PrP^C ^significantly higher than that of untreated cells (*P *< 0.01). * = amounts of PrP^C ^significantly lower than that of untreated cells (*P *< 0.01).

**Figure 7:**
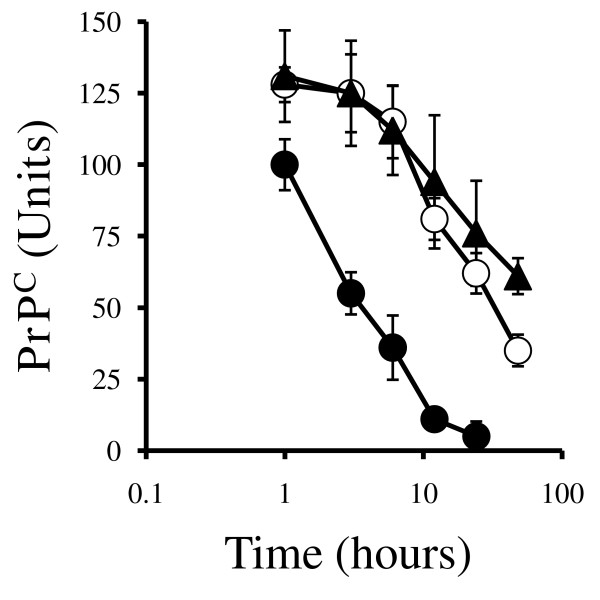
**DHA and EPA increase the half-life of surface PrP^C ^in neurons**. The amounts of biotinylated-PrP^C ^in untreated neurons (●), neurons treated with 1 μM DHA (▲) or neurons treated with 1 μM EPA (○) measured in cell extracts taken at different time points after biotinylation. Values shown are the mean average amounts of biotinylated-PrP^C ^(Units) ± SD, *n *= 9.

In a further set of experiments, treated cortical neurons were pulsed with membrane-impermeable biotin and the amounts of biotinylated-PrP^C ^in cell membranes were determined at time points thereafter. The amounts of biotinylated-PrP^C ^in neuronal extracts were reduced in a time-dependent manner. Significantly, in neurons treated with 1 μM DHA or EPA the amounts of biotinylated PrP^C ^remaining in extracts after 3, 6, 12, 24 or 48 hours were significantly greater than those in extracts from untreated cells. While the half-life of biotinylated PrP^C ^in untreated neurons was approximately 4 hours, in DHA or EPA-treated neurons the half-life was increased to approximately 24 hours (Figure 7).

## Discussion

The main goal of this study was to investigate the effects of PUFA on the production of PrP^Sc^, a biochemical marker for the infectious agent of prion diseases. We report that treatment with DHA or EPA, but not with AA, LA or LNA, consistently increased the amounts of PrP^Sc ^within three prion-infected neuronal cell lines (ScGT1, ScN2a and SMB cells). This effect of PUFA correlated with the number of unsaturated bonds in the acyl chain; increased PrP^Sc ^content was observed in ScGT1 cells treated with DHA (six unsaturated bonds) and EPA or DPA (five), while treatment with PUFA containing four or less unsaturated bonds (AA, DTA, ETA, LNA or LA) did not affect PrP^Sc ^formation. The increased PrP^Sc ^content of DHA or EPA-treated cells was accompanied by increased PrP^Sc ^content of culture supernatants, indicating that this treatment did not prevent the release of PrP^Sc ^via exosomes or cell damage.

Cholesterol synthesis inhibitors disrupt lipid rafts and reduced PrP^Sc ^formation [4,5]. More recently, observations that cholesterol synthesis inhibitors delay experimental scrapie [22,23] increased the interest in the concept of cholesterol reduction as a means of treating prion diseases. PUFA are reported to reduce cellular cholesterol and here we show that treatment with DHA and EPA significantly reduced the free cholesterol content of ScGT1 cells. However, the effects of DHA or EPA on cellular cholesterol differed from those of the HMG-CoA inhibitor simvastatin. For example, treatment with simvastatin, but not with DHA or EPA, significantly reduced total cholesterol levels. The reduction in free cholesterol levels seen in ScGT1 cells treated with DHA and EPA was accompanied by a significant increase in the amounts of cholesterol esters, and the total cholesterol content of DHA and EPA-treated cells was not significantly different from that of untreated cells. Although the increase in the PrP^Sc ^content of DHA and EPA-treated cells was accompanied by an increase in cholesterol esters, the addition of cholesterol esters did not increase PrP^Sc ^formation, suggesting that the relationship between cholesterol esters and PrP^Sc ^is casual rather than causal. Furthermore, the effects of DHA and EPA on cholesterol ester formation were independent of prion-infection; DHA and EPA increased cholesterol esters in uninfected N2a or GT1 cell lines and in primary cortical neurons (data not shown).

The amount of free cholesterol in cell membranes is partly determined by their fatty acid composition; the solubility of free cholesterol is dependent on the hydrophobic environment produced by the tight packing of saturated fatty acids [24]. The presence of double bonds in unsaturated fatty acids causes the acyl chain to be kinked; as a result, phospholipids containing PUFA cannot pack as tightly as phospholipids containing saturated fatty acids and do not sequester as much free cholesterol. Thus, the remodelling of membrane phospholipids to include increased amounts of DHA or EPA is expected to reduce the capacity of membranes to solubilise free cholesterol. The amounts of free cholesterol within cell membranes is tightly controlled [25,26] and excess free cholesterol trafficking into the endoplasmic reticulum (ER) is esterified by acyl-coenzyme A: cholesterol acyltransferase (ACAT) and stored in cytoplasmic droplets [27]. Our results are consistent with the hypothesis that treatment with DHA and EPA reduces the capacity of cell membranes to solubilise free cholesterol, resulting in increased cholesterol ester formation. This hypothesis also explains the observation that the addition of mevalonate did not reverse the effects of DHA and EPA. While mevalonate increased the amounts of free cholesterol in simvastatin-treated cells, in DHA or EPA-treated cells the newly synthesised cholesterol was not held in the membrane and was rapidly esterified. Similarly, while the addition of mevalonate reversed the effects of simvastatin on PrP^Sc ^formation, it did not reverse the effects of DHA and EPA.

So how could changes in membrane composition affect PrP^Sc ^formation? The composition of cell membranes affects the aggregation of PrP^Sc ^and fibril formation [28]. Therefore, changes in cell membranes induced by DHA and EPA may enhance the conversion of small PrP^Sc ^oligomers, that are thought to be sensitive to protease digestion (PrP^Sc^-sen) [29], into the larger fibrillar, protease-resistant deposits of PrP^Sc^. The amount of free cholesterol in cell membranes is also instrumental in the formation of lipid rafts [7]. These contain many signalling molecules, and an emerging paradigm is that of cell activation occurring as a consequence of individual rafts coalescing to form a platform capable of sustained cell activation. Therefore, changes in membrane free cholesterol caused by the incorporation of DHA or EPA into membrane phospholipids may affect lipid-raft formation and associated cellular functions [30,31]. In this study we examined the effects of PUFA treatment on PLA_2 _activity, as PLA_2 _has a critical role in PrP^Sc ^formation [14]. As activation of PLA_2 _in ScGT1 cells is cholesterol-sensitive [5], treatment with DHA or EPA was expected to reduce PLA_2 _activity. However, they significantly increased PLA_2 _activity and PGE_2 _production in ScGT1, ScN2a and SMB cells. The mechanisms by which DHA or EPA increased activation of PLA_2 _are unclear. Since DHA triggers the formation of lipid rafts [32] it is possible that treatment with DHA or EPA strengthens the association between PLA_2 _and PrP^Sc ^within a lipid raft. However, it should be noted that DHA may have other physical effects on membrane properties that are independent of cholesterol reduction [33]. For example, in some cells DHA released from membrane phospholipids by PLA_2 _is metabolised into docosanoids [34]. The effects of such DHA metabolites are only just being elucidated and the possibility that specific docosanoids affect PrP^Sc ^formation cannot be discounted. Similarly, PUFA have been reported to affect gene regulation [35] and we do not exclude the possibility that the effects of DHA or EPA on PrP^Sc ^formation result from gene activation.

We examined the possibility that the increased PrP^Sc ^production in DHA or EPA-treated cells might be a consequence of their effects on the expression of PrP^C^. Treatment of neurons with DHA or EPA did not affect the total amounts of PrP^C ^in cell extracts, or the distribution of PrP^C ^between lipid rafts and the bulk membrane. However, it significantly increased the amounts of PrP^C ^expressed at the cell surface. This effect of DHA and EPA contrasted with that of the cholesterol synthesis inhibitors simvastatin or lovastatin, which reduced surface PrP^C ^expression [21]. Prior studies indicated that PrP^C^, but not PrP^Sc^, is released from cell membranes following PI-PLC digestion [20], and in the present studies, the PrP released from ScGT1 cells was protease-sensitive, indicating that the PrP released was indeed PrP^C^. These assays were also performed on non-infected GT1 cells and cortical neurons with similar results; treatment with either DHA or EPA significantly increased the amounts of PrP^C ^at the cell surface, showing that the effects of DHA or EPA on PrP^C ^expression were not secondary to their effects on PrP^Sc ^formation.

These observations are relevant as antibody studies suggest that the conversion of PrP^C ^to PrP^Sc ^occurs at the cell surface or after internalisation from the cell surface [36]. Our hypothesis, that treatment with DHA or EPA increases the amount of PrP^C ^at the cell surface, a site where it could be readily converted to PrP^Sc^, is supported by the positive correlation obtained between the amounts of PrP^C ^at the cell surface and PrP^Sc ^in ScGT1 cells. In addition, the half-life of surface PrP^C ^was greatly increased in DHA or EPA-treated cells. Taken together these results suggest that treatment with DHA or EPA alters endocytosis and intracellular trafficking of PrP^C^, consistent with reports that the intracellular trafficking of PrP^C ^is critical for PrP^Sc ^formation [37,38]. The effect of DHA or EPA on PrP^C ^is relevant not only for prion diseases but may also have implications for Alzheimer's disease. The cellular PrP^C ^present in lipid rafts inhibits beta-secretase activity, reducing the cleavage of amyloid precursor protein and amyloid β formation [39]. Thus the regulation of PrP^C ^levels may represent an innovative approach for the modulation of amyloid deposition in the brain of individuals affected by Alzheimer's disease.

## Conclusion

We have shown that treatment of prion-infected cells with DHA or EPA alters the composition of cell membranes; it decreased amounts of free cholesterol and increased cholesterol esters. In contrast to the effects of cholesterol synthesis inhibitors, treatment with DHA or EPA increased PrP^Sc ^formation. Treatment with DHA or EPA greatly increased the expression of PrP^C ^at the cell surface, a putative site for PrP^Sc ^formation, and altered cell signalling activity within prion-infected cells, increasing the activation of PLA_2 _that is required for PrP^Sc ^formation [14]. Cholesterol depletion has been proposed as a therapeutic strategy for prion diseases, based on reports that cholesterol synthesis inhibitors reduced PrP^Sc ^formation. The studies reported here demonstrate that cholesterol depletion *per se *does not reduce PrP^Sc ^formation and cautions against such an approach. While these observations provide some insight into the composition of lipid rafts and the activation of signalling pathways that initiate the formation of PrP^Sc^, future studies are needed to examine the precise relationship between free cholesterol, PrP^C ^expression and PrP^Sc ^formation.

## Methods

### Cell lines

Prion-infected ScGT1, ScN2a or SMB neuronal cell lines were grown in Ham's F12 medium supplemented with 2 mM glutamine, 2% foetal calf serum (FCS) and standard antibiotics (100 U/ml penicillin and 100 μg/ml streptomycin). To measure the effect of PUFA on PrP^Sc ^formation, ScGT1, ScN2a or SMB cells were plated in 6-well plates (10^5 ^cells/well) and cultured in the presence of PUFA, mevalonate or simvastatin. Cells were grown with daily changes of media and the amounts of cell-associated PrP^Sc ^were evaluated after 7 days. Cells were washed twice in phosphate-buffered saline (PBS) before cell extracts were obtained. Spent medium was collected daily to see if PrP^Sc ^was released into the supernatant. Spent media was digested with proteinase K (1 μg/ml for 1 hour at 37°C); digestion was stopped with mixed protease inhibitors. The digested supernatant was concentrated by centrifugation with a 30 kDa filter and diluted to an equivalent of 10^6 ^cells/ml.

### Primary neuronal cultures

Primary cortical neurons were prepared from the brains of mouse embryos (day 15.5) after mechanical dissociation, cell sieving and isolation on histopaque (Sigma, Poole, UK). Neuronal precursors were plated (10^6 ^cells/well) in 24-well plates coated with 5 μg/ml poly-L-lysine) in Ham's F12 containing 5% FCS for 2 hours. Cultures were shaken (600 rpm for 5 minutes) and non-adherent cells removed by two washes in PBS. Neurons were grown in neurobasal medium (NBM) containing B27 components (Invitrogen, Paisley, UK) for 7 days before they were incubated with test compounds. Immunohistochemistry showed that less than 3% of cells stained for GFAP (astrocytes) or for F4/80 (microglial cells).

### Membrane extracts

Cells were homogenised in a buffer containing 10 mM Tris-HCL, 150 mM NaCl, 10 mM EDTA, 0.5% Nonidet P-40, 0.5% sodium deoxycholate, 0.2% sodium dodecyl sulphate (SDS) at 10^6 ^cells/ml. Mixed protease inhibitors (AEBSF, Aprotinin, Leupeptin, Bestain, Pepstatin A and E-46 (Sigma)) were added to some cell extracts. Membranes were prepared by repeated passage with a Wheaton homogeniser; nuclei and large fragments were removed by centrifugation (300 × *g *for 5 minutes). To determine the amount of PrP^Sc^, whole cell extract was digested with 1 μg/ml proteinase K for 1 hour at 37°C, and digestion was stopped with mixed protease inhibitors. Digestion of GT1 cell extracts with 1 μg/ml proteinase K for 1 hour at 37°C completely reduced the PrP signal; that is, complete digestion of PrP^C ^was achieved. The soluble material was split into two samples, one of which was heated to 95°C for 5 minutes and tested in a PrP-specific ELISA (see below). The other sample was mixed 1:1 with Laemmli buffer (Bio-Rad) containing β-mercaptoethanol and boiled for 5 minutes. This fraction was run on a 12% polyacrylamide gel (Invitrogen, Paisley, UK). Proteins were transferred onto a Hybond-ECL nitrocellulose membrane (Amersham Biosciences, UK) by semi-dry blotting. Membranes were blocked using 10% milk powder, and PrP was detected by incubation with a mouse monoclonal antibody (mAb) ICSM18 (D-Gen, ), followed by biotinylated rabbit anti-mouse IgG (Dako, Ely, UK) and ExtrAvidin-peroxidase (Sigma). Detection of bound antibody was by the enhanced chemiluminescence kit (Amersham Biosciences). Non-digested samples were boiled in Laemmli buffer containing β-mercaptoethanol for 5 minutes and run on a 15% polyacrylamide gel. Proteins were transferred onto a Hybond-P PVDF membrane (Amersham Biotech, UK) by semi-dry blotting. Membranes were blocked using 10% milk powder and β-actin was detected by incubation with a mouse mAb (clone AC-74, Sigma). Detection of bound antibody was by the enhanced chemiluminescence kit.

### Isolation of detergent-resistant membranes (lipid rafts)

To differentiate between the normal bulk membrane and the lipid raft micro-domains, cells were homogenised in an ice-cold buffer containing 1% Triton × 100, 10 mM Tris-HCl, 150 mM NaCl, 10 mM EDTA and mixed protease inhibitors at 10^6 ^cells/ml (as above). Membranes were prepared by repeated passage with a Wheaton homogeniser and nuclei and large fragments were removed by centrifugation (300 × *g *for 5 minutes at 4°C). The subsequent post-nuclear supernatant was incubated on ice (4°C) for 1 hour and centrifuged (16,000 × *g *for 30 minutes at 4°C). The supernatant was reserved as the normal bulk membrane while the insoluble pellet was homogenised in warm extraction buffer containing 10 mM Tris-HCL, 150 mM NaCl, 10 mM EDTA, 0.5% Nonidet P-40, 0.5% sodium deoxycholate, 0.2% SDS and mixed protease inhibitors at an equivalent of 10^6 ^cells/ml, and centrifuged (10 minutes at 16,000 × *g*); the soluble material was reserved as the lipid raft fraction.

### PrP ELISA

The amount of PrP present in cell extracts was determined in a sandwich ELISA using commercially available, defined mAbs as previously described [19]. Briefly, Nunc Maxisorb immunoplates were coated with 0.5 μg/ml mAb ICSM18 (D-Gen) which recognises amino acids 146 to 159 of murine PrP [40]. Samples were applied and detected with biotinylated mAb ICSM35 (D-Gen) (which recognises an epitope between amino acids 91 and 110) [41]. Biotinylated mAb was detected using extravidin-alkaline phosphatase and 1 mg/ml 4-nitrophenyl phosphate in a diethanolamine buffer (Sigma). Absorbance was measured on a microplate reader at 450 nm and the amount of PrP in cell extracts was calculated by reference to a standard curve of recombinant murine PrP (Prionics, Zurich, Switzerland); its limit of detection was 50 pg/ml.

### Activated cPLA_2_ ELISA

The activation of cPLA_2 _is accompanied by phosphorylation of the 505 serine residue which can be measured by phospho-specific antibodies. The amounts of activated cPLA_2 _in cells were measured by a sandwich ELISA using a capture antibody (mouse mAb anti-cPLA_2_, clone CH-7, Upstate, Milton Keynes, UK) and a rabbit polyclonal anti-phospho-cPLA_2 _(Cell Signalling Technology). Bound antibodies were detected with biotinylated anti-rabbit IgG (Dako), extravidin-alkaline phosphatase and 1 mg/ml 4-nitrophenyl phosphate in a diethanolamine buffer (Sigma). Absorbance was measured at 450 nm and the amounts of activated cPLA_2 _were calculated from a standard curve using nonlinear regression. Samples were expressed as 'units cPLA_2_' where 100 units was defined as the amount of activated cPLA_2 _in 10^6 ^untreated cells. A standard curve was generated from this sample using sequential log 2 dilutions (range 100 to 1.56 units/well).

### PGE_2 _assay

The amounts of PGE_2 _produced by cells were determined by using an enzyme-immunoassay kit (Amersham Biotech, Amersham, UK) according to the manufacturer's instructions. This assay is based on competition between unlabelled PGE_2 _in the sample and a fixed amount of labelled PGE_2 _for a PGE_2_-specific antibody. The detection limit of this assay is 20 pg/ml.

### Quantification of cell surface PrP^C^

The amounts of PrP^C ^expressed at the cell surface were determined by treating cells with 0.2 units of PI-PLC for 1 hour at 37°C (10^6 ^cells/ml). PI-PLC is cell-impermeable and acts on the GPI anchors that tether some proteins, including PrP^C^, to the cell surface. The amounts of PrP^C ^released into the culture supernatant following PI-PLC digestion were measured by PrP ELISA (as above).

### Half-life of PrP^C^

The half-life of surface PrP^C ^in neurons was determined by incubating 10^6 ^cells with PBS containing 10 μg/ml membrane-impermeable sulfo-biotin-X-NHS (Pierce, Cramlington, UK) for 10 minutes. Cells were washed four times with ice-cold PBS containing 10% FCS to remove unbound biotin and incubated in fresh NBM. Cell extracts were collected 1, 3, 6, 12, 24 and 48 hours later. The amounts of biotinylated PrP^C ^in cell extracts were measured in a modified ELISA. Maxisorb Immunoplates (Nunc, Rosklide, Denmark) were pre-coated with 10 μg/ml streptavidin (Sigma) and blocked with 10% milk powder. Samples were added for 1 hour and the amounts of bound biotinylated PrP^C ^were determined by incubation with the PrP-specific mouse mAb 4F2, anti-mouse IgG-alkaline phosphate and 1 mg/ml 4-nitrophenyl phosphate in a diethanolamine buffer. Absorbance was measured at 450 nm. The amounts of biotinylated PrP^C ^were expressed as units, where 100 units equalled the amount of biotinylated-PrP^C ^in 10^6 ^untreated neurons 1 hour after biotinylation.

### Cholesterol and protein content

Cellular cholesterol and protein content were determined in cell extracts. Protein concentrations were measured using a micro-BCA protein assay kit (Pierce, Cramlington, UK). The amounts of cholesterol were measured using the fluorometric Amplex Red cholesterol assay kit (Invitrogen), according to the manufacturer's instructions. Briefly, cholesterol is oxidised by cholesterol oxidase to yield hydrogen peroxide and ketones. The hydrogen peroxide reacts with 10-acetyl-3, 7-dihydroxyphenoxazine (Amplex Red reagent) to produce highly fluorescent resorufin, which is measured by excitation at 550 nm and emission detection at 590 nm. By performing the assay in the presence or absence of 50 units/ml cholesterol esterase and 20 μM TMP-15, which inhibits the esterification of free cholesterol, the assay can also determine the amounts of esterified cholesterol within samples.

### Reagents

AA, DHA, DPA, DTA, EPA, ETA, LA, LNA, cholesterol myristate and cholesterol arachidonate were supplied by Sigma. Stock solutions were made in chloroform/methanol mixtures and frozen at 10 mM; they were thawed and diluted on the day of use. Mevalonate and simvastatin were from Calbiochem, Nottingham, UK.

### Statistical analysis

Comparison of treatment effects was carried out using one and two-way analysis of variance techniques as appropriate. *Post hoc *comparisons of means were performed as necessary. For all statistical tests significance was set at the 1% level.

## Competing interests

The authors declare that they have no competing interests.

## Authors' contributions

CB, MT and LD were responsible for the conception, planning and performance of experiments and for writing the manuscript. MT, AW and MS contributed to the planning of experiments, interpretation of results and the writing of the manuscript. All authors read and approved the final manuscript.
